# Supplementary material for: Recombinant Mitochondrial Genomes Reveal Recent Interspecific Hybridization between Invasive Salangid Fishes
Source: Life (Basel). 2022 Apr 29;12(5):661. doi: 10.3390/life12050661 (PMC9144084; doi:10.3390/life12050661)
Supplement: Supplementary file 1 [file life-12-00661-s001.zip › Table_S1_GenBank_accession_numbers_28_Apr_2022.pdf]

Table S1. Alphabetical list of the species with GenBank accession numbers of the nucleotide sequences used in this study

Taxon	GenBank accession number	Reference
Hemisalanx brachyrostralis	KJ645979	[1]
Leucosoma chinensis	MW131880	[2]
Neosalanx anderssoni	HM106492	[3]
Neosalanx taihuensis	MH348204	[4]
Neosalanx taihuensis	MW291630	[5]
Neosalanx taihuensis	JX524196	[6]
Neosalanx tangkahkeii	KP170510	[7]
Osmerus dentex	MH370836	[8]
Plecoglossus altivelis	AB047553	[9]
Protosalanx chinensis	HM106494	[3]
Protosalanx hyalocranius	KJ499917	[10]
Protosalanx chinensis	KP306787	[11]
Protosalanx chinensis	MH330683	[12]
Protosalanx chinensis	MW291629	[5]
Retropinna retropinna	AP004108	[13]
Retropinna semoni	KX421784	[14]
Salangichthys microdon	AP004109	[13]
Salanx ariakensis	AP006231	[15]
Salanx ariakensis	KM517200	[16]
Salanx cuvieri	KJ645978	[17]

References

1. Zhang, J.; Ding, M.; Qi, J.; Chen, H.; Zhang, B. Complete mitochondrial genome of *Hemisalanx brachyrostralis* (Osteichthyes: Salangidae). *Mitochondrial DNA Part A* **2016**, *27*, 836-837, doi: 10.3109/19401736.2014.919465.

2. Xiong, F.; Zhai, D.; Yu, J.; Chen, Y.; Wang, Y.; Liu, H. The complete mitochondrial genome of the Chinese noodlefish *Leucosoma chinensis* and phylogenetic analysis of Salangidae (Osteichthyes: Osmeriformes). *Mitochondrial DNA Part B* **2021**, *6*, 176-177, doi: 10.1080/23802359.2020.1860720

3. Li, J.; Xia, R.; McDowall, R.M.; Lopez, J.A.; Lei, G.; Fu, C. Phylogenetic position of the enigmatic *Lepidogalaxias salamandroides* with comment on the orders of lower euteleostean fishes. *Mol. Phylogenet. Evol.* **2010**, *57*, 932-936.

4. Wang, Y.; Wen, H.; Yao, J.; Sun, K.; Wang, W.; Liu, H.; Yang, D.; Zhang, F.; Xiong, F. Complete mitochondrial genome of the Salangid icefish *Neosalanx taihuensis* (Actinopterygii: Osmeriformes: Salangidae). *Mitochondrial DNA Part B* **2018**, *3*, 1040-1041, doi:10.1080/23802359.2018.1511840

5. Yang, Y.; Sui, Z.; Liu, K.; Liu, Y. The complete mitochondrial DNA sequence of Linyi large icefish (*Protosalanx chinensis*). GenBank submission: 10-MAY-2021. Unpublished.

6. Zhang, J.F.; Rui, C.F. The complete mitochondrial genome of *Neosalanx taihuensis* and its phylogenetic analysis. GenBank submission: 17-OCT-2012. Unpublished.

7. Zhong, L.; Wang, M.; Li, D.; Tang, S.; Zhang, T.; Bian, W.; Chen, X. Complete mitochondrial genome of Chinese icefish *Neosalanx tangkahkeiis* (Salmoniformes, Salangidae): comparison reveals *Neosalanx taihuensis* not a valid name. *Mitochondrial DNA Part A* **2016**, *27*, 3303-3305, doi: 10.3109/19401736.2015.1015014.

8. Balakirev, E.S.; Kravchenko, A.Yu.; Romanov, N.S.; Ayala, F.J. Complete mitochondrial genome of the Arctic rainbow smelt *Osmerus dentex* (Osmeriformes, Osmeridae). *Mitochondrial DNA Part B* **2018**, *3*, 881-882.

9. Ishiguro, N.; Miya, M.; Nishida, M. Complete mitochondrial DNA sequence of ayu *Plecoglossus altivelis*. *Fish. Sci.* **2001**, *67*, 474-481.

10. Lu, C.; Gu, Y.; Li, C.; Cheng, L.; Sun, X.; Tang, F. Complete mitochondrial genome of clearhead icefish *Protosalanx hyalocranius* (Salmoniformes: Salangidae). *Mitochondrial DNA Part A* **2016**, *27*, 514-515.

11. Fu, J.; Xue, T.; Li, J. Complete mitochondrial genome of large icefish, *Protosalanx chinensis* (Salmoniformes: Salangidae). GenBank submission: 2-MAR-2015 Unpublished.

12. Liu, Y.; Song, C.; Chen, J. The mitochondrial genome of clearhead icefish *Protosalanx chinensis* (Salmoniformes: Salangidae). *Mitochondrial DNA Part B* **2018**, *3*, 774-775, doi:10.1080/23802359.2018.1491335

13. Ishiguro, N.B.; Miya, M.; Nishida, M. Basal euteleostean relationships: a mitogenomic perspective on the phylogenetic reality of the 'Protacanthopterygii'. *Mol. Phylogenet. Evol.* **2003**, *27*, 476-488.

14. Schmidt, D.J.; Islam, R.; Hughes, J.M. Complete mitogenomes for two lineages of the Australian smelt, Retropinna semoni (Osmeriformes: Retropinnidae). *Mitochondrial DNA B Resources* **2016**, *1*, 615-616.

15. Ishiguro, N.B.; Miya, M.; Inoue, J.G.; Nishida, M. *Sundasalanx* (Sundasalangidae) is a progenetic clupeiform, not a closelyrelated group of salangids (Osmeriformes): mitogenomic evidence. *J. Fish Biol.* **2005**, *67*, 561-569.

16. Zhang, H.; Xian, W. The complete mitochondrial genome of the larvae *Salanx ariakensis* (Osmeriformes, Salangidae) from Yangtze estuary. *Mitochondrial DNA Part A* **2016**, *27*, 1902-1903, doi: 10.3109/19401736.2014.971278.

17. Ding, M.; Wang, Z.; Qi, J.; Zhang, B.; Zhang, J. Mitochondrial genome of the *Salanx cuvieri* (Osteichthyes: Salangidae). *Mitochondrial DNA* **2014**, *27*, 834–835, doi:10.3109/19401736.2014.919464
