# Supplementary material for: Recombinant Mitochondrial Genomes Reveal Recent Interspecific Hybridization between Invasive Salangid Fishes
Source: Life (Basel). 2022 Apr 29;12(5):661. doi: 10.3390/life12050661 (PMC9144084; doi:10.3390/life12050661)
Supplement: Supplementary file 1 [file life-12-00661-s001.zip › Text_S1_COI_ND4_CR_Recombinant_Sequences_Alignment.pdf]

Text S1. Alignment of variable nucleotide sites of the *Protosalanx chinensis* (KJ499917, MW291629) and *Neosalanx tangkahkeii* (KP170510) full mitochondrial genomes, showing evidence of recombination at the *COI* and *ND4* genes, and also at the control region

```
---------------------------------------------------------------------------------------------------------------------
[                                                                    1111111111 1111111111 1111111111 1111111111 1111111222 22222]
[                                    12334446 7777788888 8888999999 0001111122 2344555555 5555566666 7777788888 8889022222 22233]
[                                   650582330 0399900012 2335023333 0110023314 8977001123 4456823688 1455803347 7881204677 78967]
[                                  4377095563 9204623696 8235092358 8596725665 2219265728 3472444878 3234516903 8472025757 85720]
#KJ499917_Protosalanx_chinensis    GCTGACTTAT TCAACTCCGC GTGATTTCGT AATTTTCTGG AGGAACCTTG AGTGATATAC ACCCTCACAT GAGATCGTGA GTTGC
#KP170510_Neosalanx_tangkahkeii    ATCATTCCGC CAGGTCTTAT ACCGCCCAAC GGCCCATGAA TAAGGTTACA GAAACCGCGA GTTTCTCTGC ATAGCAACAG ACCTA
#MW291629_Protosalanx_chinensis    .......... .......... .......... .........A .A........ .......... .......... .......... .....

[                                  2222222222 2222222222 2222222222 2233333333 3333333333 3333333333 3333333333 3333333333 33333]
[                                  3344466677 7777778899 9999999999 9900000000 0000001111 1111111112 2222222222 2223333333 33333]
[                                  8801256712 4688992802 2344556777 9901222335 5678990111 3455677890 0002234455 7990112344 66999]
[                                  2421357009 7329681231 7325176235 0387036253 9529251036 4925836513 4674792514 5035143214 25013]
#KJ499917_Protosalanx_chinensis    GCTAAAATAG AGTTGGATTT GTATTCATTA AATCTATGCA CGGTTCCCGC AGTCTCATTT GCCGCTAGGG TAAACCGCAG ATGTA
#KP170510_Neosalanx_tangkahkeii    ATAGG.GCGA GACCAAGCCC AACCCTGCCC GCCTCGCAAG TAACCTTTAT GACTCTGACC ATTATCCAAA .CGGTAAACA GAACG
#MW291629_Protosalanx_chinensis    .....GG... .......... .......... .......... .......... .......... .......... C......... .....

[                                  3333333333 3333333333 3333333333 3333333333 3333333333 3333333333 3333333333 3344444444 44444]
[                                  3444444444 4444445555 5555555555 6666666666 6677777777 7777777777 7777888889 9900111111 11222]
[                                  9001122334 5667990112 2333456889 1122344677 8900001122 3344445578 8999013392 4516002666 99334]
[                                  5350358146 5146170581 7069276150 4839847817 6814780958 4734695893 8147460379 6091131169 39587]
#KJ499917_Protosalanx_chinensis    CGGGTAACCG TCCGCAATAA CCTCGGACTA ATATATTTAA GATCGGCGTA GCATGGAATC CAAGTGGGTG ATGGTGGTAC CTCGT
#KP170510_Neosalanx_tangkahkeii    TAAAGGGTTA CTTATGGCTC TTCAAACTCG CCGCCCACGG CGCGAATCCG ATGCAAGGCT ATCACCACCA TCAACAACGT ACAAC
#MW291629_Protosalanx_chinensis    .......... .......... .......... .......... ....A..... .......... ....C..... .......... .....

[                                  4444444444 4444444444 4444444444 4444444444 4444444444 4444444444 4444444444 4444444444 44444]
[                                  2222223333 3333333334 4444444444 4444455555 5555555555 5555666666 6666667777 7777778888 88888]
[                                  6678880334 5567788990 0011234455 6788901223 3455566677 8899223455 5789990123 6889990112 24466]
[                                  2840397142 5846958470 3928402847 9212051031 5206925814 0625287625 8981573515 9123798570 34825]
#KJ499917_Protosalanx_chinensis    TACTTTAATC CAGGGAAATC CAGTGATTCC GGAATCAAAC AAGTAAAGTG AGTACTTTCA ATCCATTTGC ACCAACTCAG ATATA
#KP170510_Neosalanx_tangkahkeii    CCTCCCGCCT TGAAAGGCCT TCACAGCATT AACGCTCCCG G.ACGGGACA GACGTCGCAG GCTTTACGAT TATGTACT.A CCTCG
#MW291629_Protosalanx_chinensis    .......... .......... .......... .......... .G........ .......... .......... ........G. .....

[                                  4444444444 4444444455 5555555555 5555555555 5555555555 5555555555 5555555555 5555555555 55555]
[                                  8888899999 9999999900 0000000000 1111112333 4455666666 6677777777 7777788888 8888888889 99999]
[                                  7779901112 2456788902 2356677779 0124783135 2548333456 6911223444 5677900122 3455667990 01233]
[                                  1475870362 8354028440 7310602590 1194480358 3165069870 6914098147 6514245325 1625143146 98709]
#KJ499917_Protosalanx_chinensis    AACAATGCGC ACTATCTGGG ATCTATACGA CGAGTATTCT CATGCCGGTT CACAAATCCT ATCTACTTAT GCTGTATCGC CATGT
#KP170510_Neosalanx_tangkahkeii    GGTGCCCTAT GTCGCACA.. GCTCCCGTAC TAGACTCATA TGA....... ..TGCGCTTC GCACGTCCTA AACAGGGTCT TGCTC
#MW291629_Protosalanx_chinensis    .......... ........AA .......... .......... ...ATTAACA TG........ .......... .......... .....
```

```
[                                      5555555566 6666666666 6666666666 6666666666 6666666666 6666666666 6666666666 6666666666 66666]
[                                      9999999900 0000000000 0001111111 1222222333 3333333333 3344444444 4444445555 5555555555 56666]
[                                      4566789900 1345567888 8990122236 7233569001 2233556788 8801112244 4567770123 3345567899 90335]
[                                      2403516958 7270955136 9284305857 0406736587 0929365103 6973795806 9223699213 6921435401 92289]
#KJ499917_Protosalanx_chinensis        AGTCACTCAA CGCATCTTAG AGGTCTATCA ACCATCCGTA ACCTCGTTAG CCCTAATTAT AATTTATGTC TATGTTATAC TCCCC
#KP170510_Neosalanx_tangkahkeii        CACTGTCTGG TATGATCCCT CCAATCGCTG GTTCCTTACG GTTCAACCGA TTTCGGCCGC GCCCCGCACG CGGACCGCCT CTTTT
#MW291629_Protosalanx_chinensis        .......... .......... .......... .......... .......... .......... .......... .......... .....

[                                      6666666666 6666666666 6666777777 7777777777 7777777777 7777777777 7777777777 7777777777 77777]
[                                      6666778888 8888889999 9999000001 1111111222 3333333344 4444445555 5555555556 6666667777 88888]
[                                      6899354466 7778990244 4689113690 3457799778 1123457803 3478990023 3366778893 5568890157 01245]
[                                      8958125939 5687398004 7295064945 4605779173 3621033866 9821063892 5858170357 2512540248 84970]
#KJ499917_Protosalanx_chinensis        CGCATGTGAC TTAGAAGTCC CACTGGAATT TTTGATGTCT CCCTGCCCAC CCGTGCCATG TAGTGACGGC ATATTGGTCG ATTCA
#KP170510_Neosalanx_tangkahkeii        TAGGCACAGA CCGAGGACTT TGTCAAGGCC CCCAGAACTC TATCATATTT TAACATAGCA CGACAGTATT GCGCCACCTA GCCTT
#MW291629_Protosalanx_chinensis        .......... .......... .......... .......... .......... .......... .......... .......... .....

[                                      7888888888 8888888888 8888888888 8888888888 8888888888 8888888888 8888888888 8888888888 88888]
[                                      9000000000 1111111122 2222222233 3333333333 3333444444 4444444444 5555555556 6666666666 66677]
[                                      9112578889 0125677800 1123466801 1223345555 6668011233 4445567789 0134558990 0001234456 88914]
[                                      7057151490 8405704879 6843239485 7392570269 2589106512 0362873951 9205174460 2584051792 34340]
#KJ499917_Protosalanx_chinensis        CCACTAATCA TATGTTCCAA ATTTGAATTT ATTTAAGTCT GTTAGCCATT GGCCCTTTAT ACAACCCTAC AACTCTTCAC TCAAA
#KP170510_Neosalanx_tangkahkeii        TTGACCGATT CCCACATTCG GCCCAGCCCC TGCCGTACTC ACCGATAGCC AATTGCCCGC CTGCTTTCTT GGACTCCTTA ATGGG
#MW291629_Protosalanx_chinensis        .......... ...A...... .C........ .......... .......... .......... .......... .......... .....

[                                      8888888888 8888888889 9999999999 9999999999 9999999999 9999999999 9999999999 9999999999 99999]
[                                      7777777788 8899999990 0000000011 1111111122 2222222222 2333333333 3344444444 4445555555 66666]
[                                      5678899938 8913456890 1333567712 2355688912 3455677789 9000122446 7902344666 7882445778 01112]
[                                      8432801453 6894051270 8036132813 6236506535 4025436924 7479814286 5380847589 4682695065 02784]
#KJ499917_Protosalanx_chinensis        AGCTCGTAAT CCTATTTATA ATCTTATAAT CTAACCCCTT GCGTTGGGAC GGCGCGGTTA CTCAGTTTTT CGTACACCTT GCATA
#KP170510_Neosalanx_tangkahkeii        GAACTACCGC TTCG.ACGCT GCTAAGCGGC TCGGTATTCC TAACCACAGT AATAAACCCG GCTCTCCACC TAAGTGTGCC ATCCT
#MW291629_Protosalanx_chinensis        .......C.. ....A..... .......... .......... .......... .......... .......... .......... .....

[                                                                                      1111111111 1111111111 1111111111 1111111111 11111]
[                                      9999999999 9999999999 9999999999 9999999999 0000000000 0000000000 0000000000 0000000000 00000]
[                                      6666666777 7777777888 8888888899 9999999999 0000011111 1111122222 2222222333 3333333444 44444]
[                                      4678899000 4477889011 3346688900 1234445789 0115733567 7788933455 6667789111 2246899001 22445]
[                                      1190817469 5858473547 5845809214 6840395324 0254314813 4925136817 0362810147 3918912395 14281]
#KJ499917_Protosalanx_chinensis        TAAAAATGTA GCCACAGTAA ACAATCCTCA CACGGGTGTC GATAGGGTTC CACAATAACT ATCGCCGCCC ACTCATTTAT GTAGA
#KP170510_Neosalanx_tangkahkeii        CGGGTGCACG ATACTGACCG GTGGCTTCTG GCTAAACCCT AGCGAAACC. G..T...... .......... .......... .....
#MW291629_Protosalanx_chinensis        .......... .......... .......... .......... ...G.....T GTATGCCGTC CCTATAAGTT GTCTGCCCGC ACGAG
```

```
[                                  1111111111 1111111111 1111111111 1111111111 1111111111 1111111111 1111111111 1111111111 11111]
[                                  0000000000 0000000000 0000000000 0000000000 0000000000 0000000011 1111111111 1111111111 11111]
[                                  4444455555 5555666666 6666666777 7777777888 8888888899 9999999900 0000000000 1111111111 11112]
[                                  6888902333 5667011114 5777999001 1467789001 2335678911 1355667945 5677888999 0123455667 88890]
[                                  9157689039 6357503697 0036134392 5292549587 3259273513 9125170724 7925147369 5400436281 06781]
#KJ499917_Protosalanx_chinensis    ATTCGAGGGA TTGGCGCCAG GTGGGCACAC GATCCCGGGA TTCCCCAAGC CAATAGGCTA CATATCCACG CAGCTCCGCG AGGAA
#KP170510_Neosalanx_tangkahkeii    .......... .......... .......... .......... .......... .......... .......... .......... .....
#MW291629_Protosalanx_chinensis    GCCAAGATAG CCACTATTGA ACCTAGCTGT AGCTTTAAAG ACTTTTGGAT TGGGGAATCT TGCGAATGGA TGATATTCTA GAAGG

[                                  1111111111 1111111111 1111111111 1111111111 1111111111 1111111111 1111111111 1111111111 11111]
[                                  1111111111 1111111111 1111111111 1111111111 1111111111 1111111111 1111111111 1111111111 11112]
[                                  2222222222 2333333333 3344444444 4444455555 5555555555 5566666666 6666677777 7777888899 99990]
[                                  0113455578 8012236677 7900012334 5678900112 3335567778 8800012244 4678901222 3556122226 67780]
[                                  4014325832 8001707925 8925840384 6646217695 1472880362 5846981725 8051157369 2365305657 95761]
#KJ499917_Protosalanx_chinensis    TCTGCTTTCG CCTCCGCCCT CCACCAGGCA AACTCCTGAC GCGCTCACCA CTCCTGTGCC CCCACTCGCA GGTAGCCTTC GCTTT
#KP170510_Neosalanx_tangkahkeii    .......... .......... .......... .......... .......... .......... .......... ........CG ATCCC
#MW291629_Protosalanx_chinensis    ATCTTCACAT TAATTATGTC TTGTTTCAGC GTACTTAAGT ATATATGAAG TCTGCCCATT TTTGTCGATT AACGATTC.. .....

[                                  1111111111 1111111111 1111111111 1111111111 1111111111 1111111111 1111111111 1111111111 11111]
[                                  2222222222 2222222222 2222222222 2222222222 2222222222 2222222222 2222222222 2222222222 22222]
[                                  0000000000 1111111111 2222222222 2222233333 3333344444 4444455555 5566666667 7777777777 77788]
[                                  0123447788 0002236999 1233445667 7888913445 6777801222 4447812344 5813455780 1344667778 99923]
[                                  4158450328 3694860369 7328479281 7569534095 7036265147 2582113214 0034958986 2625090258 06998]
#KJ499917_Protosalanx_chinensis    ATCCGTCATC TAAGATTCTC CCTTTTGGTT GGAGGACTAA TGTTAGCACC GGTCATCCCG CAATATAAGT ATGCACCGGG GCCCT
#KP170510_Neosalanx_tangkahkeii    GCTTACAGCA CCCCGCCTCT TTCCCCAAAC A.CAAGTCGG AACCGATGTT AACTGCTTTA AGCCGCCGAC GCAAGTAAAA ATTTC
#MW291629_Protosalanx_chinensis    .......... .......... .......... .A........ .......... .......... .......... .......... .....

[                                  1111111111 1111111111 1111111111 1111111111 1111111111 1111111111 1111111111 1111111111 11111]
[                                  2222222222 2222222222 2333333333 3333333333 3333333333 3333333333 3333333333 3333333333 33333]
[                                  8888999999 9999999999 9000000000 0001111111 2222222333 3333333333 3333344444 4444444445 55555]
[                                  4557001122 2233345689 9002334455 6790112346 0133447001 1223444567 7788911234 4555578890 11223]
[                                  4397470623 5814738481 7364065818 0508470242 1357065362 8176259113 5847917327 8067981351 37592]
#KJ499917_Protosalanx_chinensis    GCGTTGTGCA TCCTGACTGA ACCCCAAAAA GTTAGGACTT ATGACCGTTC TTCCAGCGGA CTTTTTGTTG ACCATTAGAG AACTA
#KP170510_Neosalanx_tangkahkeii    CGACCAAATG CTTCAGTCAG GAAATGGGGC ACCCCAGGCG GCACTTACCT CCTTCCTAAC ACCCCCTCCT GATGCCGAGC GTTAC
#MW291629_Protosalanx_chinensis    .......... .......... .......... .......... .......... .......... .......... ......G... .....

[                                  1111111111 1111111111 1111111111 1111111111 1111111111 1111111111 1111111111 1111111111 11111]
[                                  3333333333 3333333333 3333333333 3333333333 3333333333 3333333333 3333334444 4444444444 44444]
[                                  5555555555 5556666666 6666777777 7777888888 8888888888 8999999999 9999990000 0000000000 11111]
[                                  4455566667 7780123556 7889012445 5668000011 2344455667 8000111245 6677890113 3455667789 01345]
[                                  0635814783 4929949040 5470376140 3891146739 2903912010 5039258724 7925498781 8723254738 70702]
#KJ499917_Protosalanx_chinensis    TCTGGCTAGT GCCGAATAAA GTTAGAGCAG TTCCTAGCCG AGGTCGAAAC AGGGATTAGA GTTAGAATAA TGACGATAAG AAGGA
#KP170510_Neosalanx_tangkahkeii    CTCAATCC.C ATACGGCTGC ACCGATCTGA CCT.CCATTC GAACTAGGGA GAAAGCCGAG ACCGAGGCGG CAGAACCCCA GGAAG
#MW291629_Protosalanx_chinensis    ........C. .......... .......... ...A...T.. .......... .......... .......... .......... .....
```

```
[                                   1111111111 1111111111 1111111111 1111111111 1111111111 1111111111 1111111111 1111111111 11111]
[                                   4444444444 4444444444 4444444444 4444444444 4444444444 4444444444 4444444444 4444444444 44455]
[                                   1111112222 2222223333 3444444444 4445555555 5566666666 6777777777 7777888888 8999999999 99900]
[                                   5778891122 3458890344 9001133456 6790112578 9912778899 9012333345 5889012678 8234466678 99901]
[                                   8367874814 0871502013 9254528295 8788365805 4781281703 9546258940 3098465795 8402503621 36951]
#KJ499917_Protosalanx_chinensis     GAAGGCCCAT CATGGAATCG CAATTTTATC TGGCTCACTC ATAACCCATT CACATAAGTC GCCAGGCAAG GCCCTCCTCT AGACA
#KP170510_Neosalanx_tangkahkeii     ACGAATTTGC AGCCCGTCTA AGGCCCCGCT CAATCACTCA GCGGTTTCCC TCTGCGCACG ATAGTATGCA ATTGCTTC.C GACTC
#MW291629_Protosalanx_chinensis     .......... .........A .......G.. .......... .......... .......... .....A.... ........T. .....

[                                   1111111111 1111111111 1111111111 1111111111 1111111111 1111111111 1111111111 1111111111 11111]
[                                   5555555555 5555555555 5555555555 5555555555 5555555555 5555555555 5555555555 5555555555 55555]
[                                   0000000000 0111111111 2222222222 2222333333 3333333333 3444444444 4445555666 6677777888 89999]
[                                   1344556668 8224466899 0223334566 7899022334 4445566778 9003344467 8890118255 7855666088 85567]
[                                   4247392586 9240914217 9473698413 8439236281 5783458143 2474746949 2519586524 1527267635 60715]
#KJ499917_Protosalanx_chinensis     TATCATTCGA CCTCTGCATT TTCGTTACTA TGTGAGAATC CCAACGTCGT TGAATATTCT TCTTCCAGAA TGAGATGGCG CCAGA
#KP170510_Neosalanx_tangkahkeii     CGCTCCCTAC GTAACATGCC CCTACCGGCG CCGACAGGCA TTGGTACTAC CAGGCGCCTC CTACTTG... .......... .....
#MW291629_Protosalanx_chinensis     .......... .......... .......... .......... .......... .......... .......ACG CCCCTCCCTT TGGTT

[                                   1111111111 1111111111 1111111111 1111111111 1111111111 11111]
[                                   5566666666 6666666666 6666666666 6666666666 6666666666 66666]
[                                   9900000000 0000011111 1112222223 4444455555 5666677777 77778]
[                                   8901112346 6778822368 9990023771 2378823566 7489900000 11158]
[                                   3252392431 2052909339 7892969145 9010708936 6710523579 17853]
#KJ499917_Protosalanx_chinensis     TTGCTTCACC ATCTTAAATG TTTCTAACTC -CTTATGCGA CTTGTGATGT AGCAG
#KP170510_Neosalanx_tangkahkeii     .......... .........C AAGTGGTAGT T.ACCCTACG GACCCAGCAA CAAT-
#MW291629_Protosalanx_chinensis     ACCTCCACTT CGACCCGTCC .......... CT.C...... .......... ....A
--------------------------------------------------------------------------------------------------------------------------------
```

The species name *Protosalanx hyalocranius* was used for the originally published mt genome KJ499917 [1]. However, *P. hyalocranius* is a synonym of *P. chinensis* [2]. Consequently, we use the species name *P. chinensis* for the mt genome KJ499917 to avoid any confusion. The *COI* (5585-5699), *ND4* (10173-11826), and CR (15622-16163) recombinant regions are highlighted in red bold. The recombinant regions are limited by flanking polymorphic sited and do not exactly correspond to the beginning and ending breakpoints detected by the program RDP4 [3]. Dots indicate nucleotide identity with the recombinant sequence (KJ499917). The hyphens represent deleted nucleotides. The numbers above the top sequence represent the position of segregating sites. Nucleotides are numbered from the beginning of the GenBank sequences (accession numbers see above).

References

1. Lu, C.; Gu, Y.; Li, C.; Cheng, L.; Sun, X.; Tang, F. Complete mitochondrial genome of clearhead icefish Protosalanx hyalocranius (Salmoniformes: Salangidae). *Mitochondrial DNA* **2016**, *27*, 514–515, doi:10.3109/19401736.2014.905834.

2. Zhang, J.; Li, M.; Xu, M.; Takita, T.; Wei, F. Molecular phylogeny of icefish Salangidae based on complete mtDNA cytochrome b sequences, with comments on estuarine fish evolution. *Biol. J. Linn. Soc.* **2007**, *91*, 325–340.

3. Martin, D.P.; Murrell, B.; Golden, M.; Khoosal, A.; Muhire, B. RDP4: Detection and analysis of recombination patterns in virus genomes. *Virus Evol.* **2015**, *1*, vev003, doi: 10.1093/ve/vev003.
