# Supplementary material for: Recombinant Mitochondrial Genomes Reveal Recent Interspecific Hybridization between Invasive Salangid Fishes
Source: Life (Basel). 2022 Apr 29;12(5):661. doi: 10.3390/life12050661 (PMC9144084; doi:10.3390/life12050661)
Supplement: Supplementary file 1 [file life-12-00661-s001.zip › Text_S2_ND4_Recombinant_Sequences_Alignment.pdf]

Text S2. Alignment of variable nucleotide sites of the *Protosalanx chinensis* (MH330683, MW291629) and *Neosalanx tangkahkeii* (KP170510) full mitochondrial genomes, showing evidence of recombination at the *ND4* gene

```
----------------------------------------------------------------------------------------------------------------------
[                                                           111111111 1111111111 1111111111 1111111111 1111112222 22222]
[                                    12334446 7777788888 8888899999 9000111112 2234455555 5555556666 6677777888 8888890222 22222]
[                                   650582330 0399900011 2233502333 3011002331 4897700112 3445682236 8814558033 4778812046 77789]
[                                  4377095563 9204623639 6823509235 8859672566 5221926572 8347242448 7832345169 0384720257 57857]
#MH330683_Protosalanx_chinensis    GCTGACTTAT TCAACTCCAG CGTGATTTCG TAATTTTCTG GAGGAACCTT GAGTGAATAT ACACCCTCAC ATGAGATCGT GAGTT
#KP170510_Neosalanx_tangkahkeii    ATCATTCCGC CAGGTCTTGA TACCGCCCAA CGGCCCATGA ATAAGGTTAC AGAAACTCGC GAGTTTCTCT GCATAGCAAC AGACC
#MW291629_Protosalanx_chinensis    .......... ........G. .......... .......... A.A....... ......T... .......... .......... .....

[                                  2222222222 2222222222 2222222222 2222333333 3333333333 3333333333 3333333333 3333333333 33333]
[                                  3333444666 7777777788 9999999999 9999000000 0000000011 1111111111 1222222222 2222233333 33333]
[                                  6788012567 1246889928 0223445567 7799012223 3556789901 1134556778 9000022344 5579901123 44669]
[                                  2024213570 0973296812 3173251762 3503870362 5395292510 3649258365 1346747925 1450351432 14250]
#MH330683_Protosalanx_chinensis    GCACTAAAAT AGAGTTGGAT TTGTATTCAT TAAATCTATG CACGGTTCCC GCAGTCTCAT TTGCCGCTAG GGTAAACCGC AGATG
#KP170510_Neosalanx_tangkahkeii    TA.TAGG.GC GAGACCAAGC CCAACCCTGC CCGCCTCGCA AGTAACCTTT ATGACTCTGA CCATTATCCA AA.CGGTAAA CAGAA
#MW291629_Protosalanx_chinensis    ..G....GG. .......... .......... .......... .......... .......... .......... ..C....... .....

[                                  3333333333 3333333333 3333333333 3333333333 3333333333 3333333333 3333333333 3333444444 44444]
[                                  3334444444 4444444455 5555555555 5566666666 6666777777 7777777777 7777778888 8999001111 11112]
[                                  9990011223 3456679901 1223334568 8911223446 7789000011 2233444455 7889990133 9245160026 66993]
[                                  1353503581 4651461705 8170692761 5048398478 1768147809 5847346958 9381474603 7960911311 69395]
#MH330683_Protosalanx_chinensis    TACGGGTAAC CGTCCGCAAT AACCTCGGAC TAATATATTT AAGATCGGCG TAGCATGGAA TCCAAGTGGG TGATGGTGGT ACCTC
#KP170510_Neosalanx_tangkahkeii    CGTAAAGGGT TACTTATGGC TCTTCAAACT CGCCGCCCAC GGCGCGAATC CGATGCAAGG CTATCACCAC CATCAACAAC GTACA
#MW291629_Protosalanx_chinensis    .......... .......... .......... .......... ......A... .......... ......C... .......... .....

[                                  4444444444 4444444444 4444444444 4444444444 4444444444 4444444444 4444444444 4444444444 44444]
[                                  2222222233 3333333333 3444444444 4444444555 5555555555 5555556666 6666666677 7777777788 88888]
[                                  3466788803 3455677889 9000112344 5567889012 2334555666 7788992234 5557899901 2368899901 12244]
[                                  8728403971 4258469584 7039284028 4792120510 3152069258 1406252876 2589815735 1591237985 70348]
#MH330683_Protosalanx_chinensis    GTTACTTTAA TCCAGGGAAA TCCAGTGATT CCGGAATCAA ACAAGTAAAG TGAGTACTTT CAATCCATTT GCACCAACTC AGATA
#KP170510_Neosalanx_tangkahkeii    ACCCTCCCGC CTTGAAAGGC CTTCACAGCA TTAACGCTCC CGG.ACGGGA CAGACGTCGC AGGCTTTACG ATTATGTACT .ACCT
#MW291629_Protosalanx_chinensis    .......... .......... .......... .......... ...G...... .......... .......... .......... G....

[                                  4444444444 4444444444 5555555555 5555555555 5555555555 5555555555 5555555555 5555555555 55555]
[                                  8888888999 9999999999 0000000000 0011111123 3344556666 6666777777 7777777888 8888888888 89999]
[                                  6677799011 1224567889 0223566777 7901247831 3525483334 5669112234 4456779001 2234556679 90012]
[                                  2514758703 6283540284 4073106025 9011944803 5831650698 7069140981 4765142453 2516251431 46987]
#MH330683_Protosalanx_chinensis    TAAACAATGC GCACTATCTG GGATCTATAC GACGAGTATT CTCATGTTAA CATACAAATC CTATCTACTT ATGCTGTATC GCCAT
#KP170510_Neosalanx_tangkahkeii    CGGGTGCCCT ATGTCGCACA ..GCTCCCGT ACTAGACTCA TATGA.CCGG TTC.TGCGCT TCGCACGTCC TAAACAGGGT CTTGC
#MW291629_Protosalanx_chinensis    .......... .......... AA........ .......... .....A.... ...G...... .......... .......... .....
```

```
[                                  5555555555 6666666666 6666666666 6666666666 6666666666 6666666666 6666666666 6666666666 66666]
[                                  9999999999 0000000000 0000011111 1112222223 3333333333 3333444444 4444444455 5555555555 55566]
[                                  3345667899 0013455678 8889901222 3672335690 0122335567 8888011122 4445677701 2333455678 99903]
[                                  0924035169 5872709551 3692843058 5704067365 8709293651 0369737958 0692236992 1369214354 01922]
#MH330683_Protosalanx_chinensis    GTAGTCACTC AACGCATCTT AGAGGTCTAT CAACCATCCG TAACCTCGTT AGCCCTAATT ATAATTTATG TCTATGTTAT ACTCC
#KP170510_Neosalanx_tangkahkeii    TCCACTGTCT GGTATGATCC CTCCAATCGC TGGTTCCTTA CGGTTCAACC GATTTCGGCC GCGCCCCGCA CGCGGACCGC CTCTT
#MW291629_Protosalanx_chinensis    .......... .......... .......... .......... .......... .......... .......... .......... .....

[                                  6666666666 6666666666 6666667777 7777777777 7777777777 7777777777 7777777777 7777777777 77777]
[                                  6666667788 8888888899 9999990000 0111111112 2233333333 4444444455 5555555555 5666666677 77888]
[                                  3568993544 6677789902 4446891136 9034577997 7811234578 0334789900 2333667788 9355688901 57012]
[                                  8989581259 3956873980 0472950649 4546057791 7336210338 6698210638 9258581703 5725125402 48849]
#MH330683_Protosalanx_chinensis    CCCGCATGTG ACTTAGAAGT CCCACTGGAA TTTTTGATGT CTCCCTGCCC ACCCGTGCCA TGTACTGACG GCATATTAGT CGATT
#KP170510_Neosalanx_tangkahkeii    TTTAGGCACA GACCGAGGAC TTTGTCAAGG CCCCCAGAAC TCTATCATAT TTTAACATAG CACGACAGTA TTGCGCC.CC TAGCC
#MW291629_Protosalanx_chinensis    .......... .......... .......... .......... .......... .......... ....G..... .......G.. .....

[                                  7777788888 8888888888 8888888888 8888888888 8888888888 8888888888 8888888888 8888888888 88888]
[                                  8899900000 0000111111 1122222222 2233333333 3333333344 4444444444 4444555555 5556666666 66666]
[                                  4545911257 8889012567 7800112346 6801122334 5555666801 1233444556 7789013455 8990000123 44568]
[                                  7062705715 1490840570 4879684323 9485739257 0269258910 6512036287 3951920517 4460258405 17923]
#MH330683_Protosalanx_chinensis    CAGCCCACTA ATCATATGTT CCAAATTTGA ATTTATTTAA GTCTGTTAGC CATTGGCCCT TTATACAACC CTACAACTCT TCACT
#KP170510_Neosalanx_tangkahkeii    TTTGTTGACC GATTCCCACA TTCGGCCCAG CCCCTGCCGT ACTCACCGAT AGCCAATTGC CCGCCTGCTT TCTTGGACTC CTTAA
#MW291629_Protosalanx_chinensis    ..TG...... .......A.. .....C.... .......... .......... .......... .......... .......... .....

[                                  8888888888 8888888888 8889999999 9999999999 9999999999 9999999999 9999999999 9999999999 99999]
[                                  6677777777 7788889999 9990000000 0011111111 1122222222 2222233333 3333334444 4444444555 55556]
[                                  8914567889 9938891345 6890133356 7712235568 8912345567 7789900012 2446790234 4666788244 57780]
[                                  4340843280 1453689405 1270803613 2813623650 6535402543 6924747981 4286538084 7589468269 50650]
#MH330683_Protosalanx_chinensis    CAAAAGCTCG TAATCCTATT TATAATCTTA TAATCTAACC CCTTGCGTTG GGACGGCGCG GTTACTCAGT TTTTCGTACA CCTTG
#KP170510_Neosalanx_tangkahkeii    TGGGGAACTA CCGCTTCG.A CGCTGCTAAG CGGCTCGGTA TTCCTAACCA CAGTAATAAA CCCGGCTCTC CACCTAAGTG TGCCA
#MW291629_Protosalanx_chinensis    .......... .C......A. .......... .......... .......... .......... .......... .......... .....

[                                                                                111111 1111111111 1111111111 1111111111 11111]
[                                  9999999999 9999999999 9999999999 9999999999 9999000000 0000000000 0000000000 0000000000 00000]
[                                  6666666666 6777777777 7888888888 8899999999 9999000001 1111111112 2222222222 2333333333 34444]
[                                  1112467889 9000447788 9011334668 8900123444 5789011573 3567778893 3455666778 9111224689 90012]
[                                  2784119081 7469585847 3547584580 9214684039 5324025431 4813492513 6817036281 0147391891 23951]
#MH330683_Protosalanx_chinensis    CATATAAAAA TGTAGCCACA GTAAACAATC CTCACACGGG TGTCGATAGG GTTCCACAAT AACTATCGCC GCCCACTCAT TTATG
#KP170510_Neosalanx_tangkahkeii    TCCTCGGGTG CACGATACTG ACCGGTGGCT TCTGGCTAAA CCCTAGCGAA ACC.G..T.. .......... .......... .....
#MW291629_Protosalanx_chinensis    .......... .......... .......... .......... .......G.. ...TGTATGC CGTCCCTATA AGTTGTCTGC CCGCA
```

```
[                                  1111111111 1111111111 1111111111 1111111111 1111111111 1111111111 1111111111 1111111111 11111]
[                                  0000000000 0000000000 0000000000 0000000000 0000000000 0000000000 0011111111 1111111111 11111]
[                                  4444444445 5555555566 6666666666 6777777777 7888888888 8899999999 9900000000 0000111111 11111]
[                                  2445688890 2333566701 1114577799 9001146778 9001233567 8911135566 7945567788 8999012345 56678]
[                                  4281915768 9039635750 3697003613 4392529254 9587325927 3513912517 0724792514 7369540043 62810]
#MH330683_Protosalanx_chinensis    TAGAATTCGA GGGATTGGCG CCAGGTGGGC ACACGATCCC GGGATTCCCC AAGCCAATAG GCTACATATC CACGCAGCTC CGCGA
#KP170510_Neosalanx_tangkahkeii    .......... .......... .......... .......... .......... .......... .......... .......... .....
#MW291629_Protosalanx_chinensis    CGAGGCCAAG ATAGCCACTA TTGAACCTAG CTGTAGCTTT AAAGACTTTT GGATTGGGGA ATCTTGCGAA TGGATGATAT TCTAG

[                                  1111111111 1111111111 1111111111 1111111111 1111111111 1111111111 1111111111 1111111111 11111]
[                                  1111111111 1111111111 1111111111 1111111111 1111111111 1111111111 1111111111 1111111111 11111]
[                                  1112222222 2222233333 3333334444 4444444445 5555555555 5555556666 6666666667 7777777788 88999]
[                                  8890011345 5578801223 6677790001 2334567890 0112333556 7778880001 2244467890 1222355612 22266]
[                                  6781401432 5832800170 7925892584 0384664621 7695147288 0362584698 1725805115 7369236530 56579]
#MH330683_Protosalanx_chinensis    GGAATCTGCT TTCGCCTCCG CCCTCCACCA GGCAAACTCC TGACGCGCTC ACCACTCCTG TGCCCCCACT CGCAGGTAGC CTTCG
#KP170510_Neosalanx_tangkahkeii    .......... .......... .......... .......... .......... .......... .......... .......... ..CGA
#MW291629_Protosalanx_chinensis    AAGGATCTTC ACATTAATTA TGTCTTGTTT CAGCGTACTT AAGTATATAT GAAGTCTGCC CATTTTTGTC GATTAACGAT TC...

[                                  1111111111 1111111111 1111111111 1111111111 1111111111 1111111111 1111111111 1111111111 11111]
[                                  1112222222 2222222222 2222222222 2222222222 2222222222 2222222222 2222222222 2222222222 22222]
[                                  9990000000 0000111111 1111222222 2222222223 3333333334 4444444445 5555556666 6667777777 77777]
[                                  7780012344 7788000223 6999123344 5667788891 3445677780 1222444781 2344581345 5780134466 77789]
[                                  5761415845 0328369486 0369732847 9281756953 4095703626 5147258211 3214003495 8986262509 02580]
#MH330683_Protosalanx_chinensis    CTTTATCCGT CATCTAAGAT TCTCCCTTTT GGTTGGAGGA CTAATGTTAG CACCGGTCAT CCCGCAATAT AAGTATGCAC CGGGG
#KP170510_Neosalanx_tangkahkeii    TCCCGCTTAC AGCACCCCGC CTCTTTCCCC AAACA.CAAG TCGGAACCGA TGTTAACTGC TTTAAGCCGC CGACGCAAGT AAAAA
#MW291629_Protosalanx_chinensis    .......... .......... .......... .....A.... .......... .......... .......... .......... .....

[                                  1111111111 1111111111 1111111111 1111111111 1111111111 1111111111 1111111111 1111111111 11111]
[                                  2222222222 2222222222 2222233333 3333333333 3333333333 3333333333 3333333333 3333333333 33333]
[                                  7788888899 9999999999 9999900000 0000000111 1111222222 2333333333 3333333334 4444444444 44455]
[                                  9923455700 1122223334 5689900233 4455679011 2346013344 7001122344 4567778891 1234455557 88901]
[                                  6998439747 0623581473 8481736406 5818050847 0242135706 5362817625 9113584791 7327806798 13513]
#MH330683_Protosalanx_chinensis    CCCTGCGTTG TGCATCCTGA CTGAACCCCA AAAAGTTAGG ACTTATGACC GTTCTTCCAG CGGACTTTTT GTTGACCATT AGAGA
#KP170510_Neosalanx_tangkahkeii    TTTCCGACCA AATGCTTCAG TCAGGAAATG GGGCACCCCA GGCGGCACTT ACCTCCTTCC TAACACCCCC TCCTGATGCC GAGCG
#MW291629_Protosalanx_chinensis    .......... .......... .......... .......... .......... .......... .......... .......... G....

[                                  1111111111 1111111111 1111111111 1111111111 1111111111 1111111111 1111111111 1111111111 11111]
[                                  3333333333 3333333333 3333333333 3333333333 3333333333 3333333333 3333333333 4444444444 44444]
[                                  5555555555 5555555666 6666666677 7777777788 8888888888 8888899999 9999999999 0000000000 00001]
[                                  1223445556 6667778012 3556788901 2445566800 0011234445 5667800011 1245667789 0113345566 77890]
[                                  7592063581 4783492994 9040547037 6140389114 6739290391 2010503925 8724792549 8781872325 47387]
#MH330683_Protosalanx_chinensis    ACTATCTGGC TAGTGCCGAA TAAAGTTAGA GCAGTTCCTA GCCGAGGTCG AAACAGGGAT TAGAGTTAGA ATAATGACGA TAAGA
#KP170510_Neosalanx_tangkahkeii    TTACCTCAAT CC.CATACGG CTGCACCGAT CTGACCT.CC ATTCGAACTA GGGAGAAAGC CGAGACCGAG GCGGCAGAAC CCCAG
```

```
#MW291629_Protosalanx_chinensis    .......... ..C....... .......... .......A.. .T........ .......... .......... .......... .....

[                                  1111111111 1111111111 1111111111 1111111111 1111111111 1111111111 1111111111 1111111111 11111]
[                                  4444444444 4444444444 4444444444 4444444444 4444444444 4444444444 4444444444 4444444444 44444]
[                                  1111111111 2222222222 3333344444 4444445555 5555556666 6666677777 7777777788 8888899999 99999]
[                                  1345577889 1122345889 0344900113 3566790112 5678991277 8899901233 3345588902 5678823446 66789]
[                                  0702836787 4814087150 2013925452 8958788365 8705478128 1703954625 8940309845 8795840250 36213]
#MH330683_Protosalanx_chinensis    AGGAGAAGGC CCATCATGGA ATCGCAATTT TTCTGGCTCA CGTCATAACC CATTCACATA AGTCGCCAGC AAAGGCCCTC CTTTA
#KP170510_Neosalanx_tangkahkeii    GAAGACGAAT TTGCAGCCCG TCTAAGGCCC CCTCAATCAC TACAGCGGTT TCCCTCTGCG CACGATAGTT GGCAATTGCT TCCCG
#MW291629_Protosalanx_chinensis    .......... .......... ...A...... .......... .A........ .......... .......... G......... .....

[                                  1111111111 1111111111 1111111111 1111111111 1111111111 1111111111 1111111111 1111111111 11111]
[                                  4455555555 5555555555 5555555555 5555555555 5555555555 5555555555 5555555555 5555555555 55555]
[                                  9900000000 0000011111 1111222222 2222222233 3333333333 3333344444 4444444555 5666666677 77777]
[                                  9901134455 6668822446 6899022333 4566789902 2334444556 6778900334 4467889011 8255788923 44455]
[                                  6951424739 2586924091 4217947369 8413843923 6281578345 8143247474 6949251958 6524145082 67926]
#MH330683_Protosalanx_chinensis    GACATATCAT TCGACCTCTG CATTTTCGTT ACTATGTGAG AATCCCAACG TCGTTGAATA TTCTTCTTCC AGAACCCTTC CACTC
#KP170510_Neosalanx_tangkahkeii    ACTCCGCTCC CTACGTAACA TGCCCCTACC GGCGCCGACA GGCATTGGTA CTACCAGGCG CCTCCTACTT G...TTGA-- TGGAT
#MW291629_Protosalanx_chinensis    .......... .......... .......... .......... .......... .......... .......... .ACG.T..CT ...CT

[                                  1111111111 1111111111 1111111111 1111111111 1111111111 1111111111 1111111111 ]
[                                  5555555555 5555555566 6666666666 6666666666 6666666666 6666666666 6666666666 ]
[                                  7777778888 8899999900 0000000000 0111111112 2222234444 4555555666 6777777777 ]
[                                  5666880068 8855678901 1123466778 8223679990 0237712337 8235667489 9000001115 ]
[                                  7267041453 5607153252 3924312052 9093347892 9691459021 7089366710 5235791785 ]
#MH330683_Protosalanx_chinensis    -TCCTGGTAT TTGGTTACCT CCACTTCGAC CCGTCATTTC TAACTCCTTT ATGCGACTTG TGATGTAGCA
#KP170510_Neosalanx_tangkahkeii    GATG-----C GCCAGATTGC TTCACCATCT TAAATTAAGT GGTAGTTCGA CCTACGGACC CAGCAACAAT
#MW291629_Protosalanx_chinensis    C...CCCCG. .......... .......... .....T.... ........G. .......... ..........
------------------------------------------------------------------------------------------------------------------------
```

The *ND4* (10173-11826) recombinant regions is highlighted in red bold. Other comments, see the Text S1.
