# Supplementary material for: Recombinant Mitochondrial Genomes Reveal Recent Interspecific Hybridization between Invasive Salangid Fishes
Source: Life (Basel). 2022 Apr 29;12(5):661. doi: 10.3390/life12050661 (PMC9144084; doi:10.3390/life12050661)
Supplement: Supplementary file 1 [file life-12-00661-s001.zip › Text_S3_ND5_Recombinant_Sequences_Alignment.pdf]

Text S3. Alignment of variable nucleotide sites of the *Protosalanx chinensis* (HM106494, MW291629) and *Neosalanx anderssoni* (HM106492) full mitochondrial genomes, showing evidence of recombination at the *ND5* genes.

```
[                                               111111 1111111111 1111122222 2222222222 2222333333 3333333333 3333333333 33333]
[                                    124445666 7888023334 4555566688 8888902223 4466677899 9999000000 1111111112 2223333333 33334]
[                                    512266024 6123049992 7123338811 3678424678 1258945845 7788456789 0145588891 2440244466 77891]
[                                   4754548384 6383852350 5628937847 7348216883 2560284710 2403987084 0281747801 3144203914 09512]
#HM106494_Protosalanx_chinensis     GTTATATTGT CAGGAGGTTG AATGACACCC AATGATGTAG ACAAAAACAT TATACCGCTC CGATATCCTC GAGAGAGCAT TACCT
#HM106492_Neosalanx_anderssoni      ACCGCGCCTC GGAAG..ACA GGCAGTGG.. CGAAGCACGA GT.GGGGTCC CCCGAT.TCA AC.CGATTGT TCAGAGATGA CGTTA
#MW291629_Protosalanx_chinensis     ...G...... .G...AA... ........AT .......... ..G....... ......A... ..G....... .......... .....

[                                   3333333333 3333333333 3333333333 3333333444 4444444444 4444444444 4444444444 4444444444 44444]
[                                   4444444555 5555566666 7777778888 8888999011 1222222223 3333344444 4455555555 5566666667 77788]
[                                   2245689012 3788911348 0357990123 4447244923 9012467880 3678900244 6701134566 7712345563 66811]
[                                   4754313270 5769579765 0078365198 1784859305 8138973286 3351328317 5170641017 0357654742 08946]
#HM106494_Protosalanx_chinensis     AAGTCCAGGA TAAATTAATG TCGTAGAAGC AGAAGATCGC TACGTACTTA TGGTACCGTT AGAACAAAAG TGCTTTAAAA GAACG
#HM106492_Neosalanx_anderssoni      GGACTAGA.C CCGCCCGCCC CTACGAGCAT GAGGATCTAT CGAACGTCCG CAACGT.ACC GCGCTG.CGC CATAGCGGGG AGGTA
#MW291629_Protosalanx_chinensis     ........A. .......... ..A....C.. ...G...... .......... ......A... ......GC.. .......... .....

[                                   4444444445 5555555555 5555555555 5555555555 5555555555 5555555566 6666666666 6666666666 66666]
[                                   8888889990 0000000000 0000133566 6666777777 7888888888 8999999900 0000011222 2233333444 55555]
[                                   3458990340 1115566777 7889157424 5599012245 7022336677 9011248935 6889912222 3500138779 34455]
[                                   1888479993 1797989147 8062074597 3958132505 0617093625 0217019518 4581791369 5617915289 21403]
#HM106494_Protosalanx_chinensis     TCGAAAGCAG ACGATATACA GCTCGTTTTA CACACAATCA CGACGTAATG TTAAGAATGT CGAGGTACTC ATTTACCTAA CTGGT
#HM106492_Neosalanx_anderssoni      AGTGTCATG. GA.GCGCGTG ATCAAAAACG TCT.TGGCTG AAGAACTGCA CCGCACGCAC TTGCACGTCT CCCCGTTAGG GAAAC
#MW291629_Protosalanx_chinensis     .........A ..A....... .......... ...G...... .......... CC........ .......... .......... .....

[                                   6666666666 6666666667 7777777777 7777777777 7777777777 8888888888 8888888888 8888888888 88888]
[                                   5555566667 7788888890 0011233334 4445555555 5666777899 0001111222 2222333333 3333344444 45555]
[                                   7889934783 5946679902 3979823360 1780456789 9669125346 5781466001 2468012233 3456835678 92333]
[                                   4090843383 1945872871 0366873965 7104354094 7363196155 6793869687 3783742814 7687501684 30578]
#HM106494_Protosalanx_chinensis     AAACTGATGT GGCTCAAAGT CTATGTAGCA GGTGTGCGAG GCAATCCCGC TTAACTTAAT TCATTTTTTA AATTGTCTTA ATTTT
#HM106492_Neosalanx_anderssoni      GGGTCT.CCC AA.CGGGGAC TCCCACGATC AACACCGAGA ATGGCTTT-T CCTCTCACGC CTCCCCACCG GGCCACTCCG GCCGA
#MW291629_Protosalanx_chinensis     ......G... ..T....... T......... ......G... ...G....T. .........C .......... .......... ....G

[                                                                                                                        1111]
[                                   8888888888 8888888888 8888899999 9999999999 9999999999 9999999999 9999999999 9999999999 90000]
[                                   5555666666 6677777778 8999900011 1122222223 3333333333 3333444444 4567777888 8899999999 90000]
[                                   4689000115 8815566891 3335737812 6701235660 0224777788 8899123366 7410248015 6800223478 90002]
[                                   4533147038 2831439701 4671854092 7302404363 8037578923 4929054748 9565640465 7836489821 32382]
#HM106494_Protosalanx_chinensis     ACCTAACATA TAAAAGCCTC ACCACTCACT CCTTTGTTTG GGGTACGCAC GATAACTGTT ACATTGCTAA TCAACAGAGT CACGT
#HM106492_Neosalanx_anderssoni      CTTCGGGGAT GGGGGATTCT GTTGTATGTC TTACC.CCCA AAACTACGTG CGCGGTCCAC GTCCCAACCG CTGGTGA.AC TGTAC
#MW291629_Protosalanx_chinensis     .......... .......... ......T... .....A.... .......... .......... .......... .......G.. .....
```

```
[                                    1111111111 1111111111 1111111111 1111111111 1111111111 1111111111 1111111111 1111111111 11111]
[                                    0000000000 0000000000 0000000000 0000000000 0000001111 1111111111 1111111111 1111111111 11111]
[                                    0001112222 2222223334 4444555666 6677777788 8889990000 0000000011 1111111122 2222222333 33444]
[                                    2375890234 5577882344 4559069158 8900112301 2391892445 5567888901 4556888901 1334688227 89155]
[                                    3347192354 0312032734 7068127801 5258170576 8732136170 3681369246 9351258730 5032917091 38358]
#HM106494_Protosalanx_chinensis      AACTAGGACG TAACACGACA AGGAGCAGGC TGAGTAATAG CCCTCGACGC TTGCATAGTC AATCGGTGAC AATTGTTTAT CTTGA
#HM106492_Neosalanx_anderssoni       GGTCCAACTA GGGTGTAGTG GAACATGACT CCGACCGCGA GTTCTAGTAT ACAGCCTACA GGCTA.CAGT GGCCACCCGC TCCAG
#MW291629_Protosalanx_chinensis      .......... .......... .......... .......... .......... .......... .....A.... .......... .....

[                                    1111111111 1111111111 1111111111 1111111111 1111111111 1111111111 1111111111 1111111111 11111]
[                                    1111111111 1111111111 1111111111 1112222222 2222222222 2222222222 2222222222 2222222222 22222]
[                                    4444555555 5566666666 6677777788 9990000000 0011222222 2333333334 4444455555 5666777778 88999]
[                                    6788000166 7800234456 6912234636 2270133447 8926124557 8356667880 4445701124 5158347890 45023]
[                                    4358069909 2158964792 5860540446 3463007342 7232323281 4943692175 1479483680 8277541055 38619]
#HM106494_Protosalanx_chinensis      CACCTAAATG AGGCACTTTG CCGCTAAGAA GTTATTCGTA CGGTACTTGT GTAGTGTAAG AGTTCAATAC AAAGTTGCCT GGGCA
#HM106492_Neosalanx_anderssoni       GGTGCCGGCA CAATGACCCA TACTCGGAGG ACCGCCTACG T.CCGTCGAC .CGACACGGA GACCTGGCGT GGTAC.CTTC AACTC
#MW291629_Protosalanx_chinensis      .......... .......... .......... .....C.... .A........ A......... G......... .....C.... .....

[                                    1111111111 1111111111 1111111111 1111111111 1111111111 1111111111 1111111111 1111111111 11111]
[                                    2222233333 3333333333 3333333333 3333333333 3333333333 3333333333 3333333333 3334444444 44444]
[                                    9999900000 0001111222 2222333333 3344444444 4555555555 5666666667 7777778888 8990000000 00001]
[                                    5599923455 5561156000 3478224468 8911145557 7002223566 9013778890 0012272245 9060133345 56672]
[                                    4716730703 6983621016 6847361403 6836962683 9064789237 2818476922 3492561481 6884701762 51431]
#HM106494_Protosalanx_chinensis      TTCGATAGGG TATCTTCGAA CCAGCTCAAT TTAGGGAATA AGACCTTTTG AGATGATATG CCCGGTAACA GGTCAAGTGC AGATC
#HM106492_Neosalanx_anderssoni       C.T.T.G... .......... .........C CCGTAACGGG GAGTTGCCCA TAGCACCGCA TATACCGCTG AAATCGACCT GACCT
#MW291629_Protosalanx_chinensis      CCTATCGAAA CGCGGCTAGC ATGATCAGG. .......... .........C .....C.... .......... .......... .....

[                                    1111111111 1111111111 1111111111 1111111111 1111111111 1111111111 1111111111 1111111111 11111]
[                                    4444444444 4444444444 4444444444 4444444444 4444455555 5555555555 5555555555 5555555555 55555]
[                                    1111111122 2334444555 5566677777 7788888888 9999900000 0111111222 2233333344 4444444444 66666]
[                                    2234678812 6041359336 8924812233 7701227778 3668945555 6144578258 8912446800 0223446677 46667]
[                                    3455656743 8124862474 2118476925 1410253462 9364010369 5669292781 4073142914 7891364739 97890]
#HM106494_Protosalanx_chinensis      GCAACAAGCT AAGGATTCGG CAATAATTAA ACGGCCACAG CTCCATACTT GGTTTACCTG GTAAACGTAG ACGATTCCGT CCCGC
#HM106492_Neosalanx_anderssoni       ATGGGGGATA GTAAGCCTAA TGGCCCCGGG GTAATTGTGC GCGAGCCTCC ACCCCGTGCT TCGGGGACGC GTATCCTTCA GTTCG
#MW291629_Protosalanx_chinensis      A.....G... ..A......A .......... .......... .......... .......... ........GA .......... .....

[                                    1111111111 1111111111 1111111111 1111111111 1111111111 1111111111 ]
[                                    5555555555 5555555555 5555566666 6666666666 6666666666 6666666666 ]
[                                    6677777778 8888999999 9999900000 0011111222 3444555555 5566677777 ]
[                                    7822448990 0116122233 3356911333 6812469034 0227113556 8845800156 ]
[                                    2759591781 8462023901 2943958679 6550593449 3455783231 0421846808 ]
#HM106494_Protosalanx_chinensis      TTTCTTGCGT TCCAATATTT TTGAACAACT GGCTCATTAG CCTTCGGAGA CCTTTTAGAT
```

```
#HM106492_Neosalanx_anderssoni     AG-----TAC GTT-CCCCCC CAAGCTCGGC AAAAG.AGCC .TCCAACGAG ATGCCATATC
#MW291629_Protosalanx_chinensis    ..CTCCC.CC ...G...... .......... .....T...C T..C...... ..........
--------------------------------------------------------------------------------------------------------------------------------
```

The ND5 recombinant region(12957-13360) is highlighted in red bold. Other comments, see the Text S1.
